# Magnolol Attenuates Cisplatin-Induced Muscle Wasting by M2c Macrophage Activation

**DOI:** 10.3389/fimmu.2020.00077

**Published:** 2020-02-07

**Authors:** Chanju Lee, Hyunju Jeong, Hyunji Lee, Minwoo Hong, Seon-young Park, Hyunsu Bae

**Affiliations:** Department of Physiology, College of Korean Medicine, Kyung Hee University, Seoul, South Korea

**Keywords:** sarcopenia, muscle atrophy, cisplatin, magnolol, M2c macrophages

## Abstract

Cancer chemotherapy induces sarcopenia, which is a rapid loss of muscle mass that directly restricts daily activities and leads to poor quality of life and increased mortality. Although hormone-related therapies have been used to improve appetite and nutritional status, current treatments are considered palliative. Thus, the protection of skeletal muscle loss without adverse effects is essential to allow the maintenance of chemotherapy in cancer patients. Magnolol from *Magnolia officinalis* has several pharmacological effects including anti-cancer and anti-inflammatory activities, but the protection from muscle atrophy is not well-understood. In the present study, we investigated the effects of magnolol on muscle wasting and macrophage subtypes in a cisplatin-induced sarcopenia mouse model. We showed that magnolol significantly attenuated the body weight and the muscle loss induced by cisplatin injection. The diameter of the tibialis anterior muscle was markedly increased after magnolol treatment in cisplatin-treated mice. Importantly, magnolol increased macrophage infiltration into skeletal muscle while not affecting proliferation of macrophages. Magnolol attenuated the imbalance of M1/M2c macrophages by increasing CD206^+^CD163^+^ M2c tissue reparative macrophages. Further, magnolol increased insulin-like growth factor (IGF)-1 expression. This effect was also observed in bone marrow-derived macrophages upon magnolol treatment. Taken together, magnolol may be a promising chemoprotective agent for the prevention of muscle atrophy through the upregulating M2c macrophages, which are a major source of IGF-1.

## Introduction

Sarcopenia, a pivotal feature of cancer cachexia, is defined as degenerative skeletal muscle loss and decline of muscle strength (1). Cancer chemotherapy is still regarded as a successful treatment in cancer patients as a standard care, but it has been associated with higher incidence of rapid muscle protein breakdown, which restricts daily activities and leads to poor clinical outcome, poor quality of life, and increased mortality (2). Furthermore, chemotherapies trigger nuclear factor kappa-B (NF-κB) activation (3, 4), which directly increases proteolysis and the release of inflammatory mediators in early phase (5, 6). As sarcopenia occurs rapidly and is often irreversible in late stages, there is a need to develop novel treatments for protection of cancer patients.

After muscle injury, the tissue microenvironment becomes rich with inflammatory signals from activated immune cells. The recruited cells propagate the inflammation and induce muscle cell apoptosis. Among the immune cells, macrophages in muscles play a central role in the activation and protection of myofibers after muscle inflammation and injury (7). Macrophages are often classified as M1 or M2 types, although the phenotypes of macrophages are heterogeneous in various tissue and environments (8). In early phase of damaged muscle, a large amount of inflammatory cytokines; i.e., tumor necrosis factor (TNF)-α, interleukin (IL)-1β, or (IL-6); inducible nitric oxide synthase (iNOS); and reactive oxygen species which are associated with the acceleration of myofiber lysis and protein degradation (9, 10) are secreted by pro-inflammatory M1 macrophages (11). On the other hand, alternatively activated M2 macrophages at the injury site are more abundant during the late phase of tissue repair. M2 macrophages have been known to repress the excessive inflammatory response and promoting myogenesis by producing transforming growth factor (TGF)-β and IL-10, although the role of TGF- β on muscle repair is controversial (12–14). Further, M2c macrophages defined as CD163-expressing macrophages sustain muscle healing and are regarded as an important source of insulin-like growth factor (IGF)-1, which mediates muscle cell proliferation, differentiation, and the survival (15, 16). Thus, M1 and M2 macrophage balance is critical in muscle protection (15, 17).

Magnolol (5,5′-diallyl-2,2′-dihydroxybiphenyl), one of the active components of *Magnolia officinalis* extracts, is lipophilic and has a hydroxylated biphenoid structure. Magnolol has several pharmacological effects, including anti-cancer, anti-oxidant, anti-microbial, and anti-inflammatory effects (18–23). Magnolol was reported to directly ameliorate muscle atrophy by inactivating myostatin and *Foxo3* signaling (24). However, the correlations with macrophage infiltration upon magnolol treatment in muscle atrophy are not well-understood, although magnolol exhibits anti-inflammation activity and inhibits lipopolysaccharide (LPS)-activated M1 macrophages through the inhibition of NF-κB activation signaling (25, 26).

Here, we investigated the effects of magnolol on muscle wasting in a chemotherapy-induced muscle wasting mouse model. We further studied the changes of macrophage subtypes induced by magnolol on pro-repair CD163^+^ M2c macrophages. Our results show that the modulation of macrophages in muscle tissue may represent a novel therapeutic approach in cancer patients to prevent the dose-limiting side effects of anti-cancer agents.

## Materials and Methods

### Chemicals

Cisplatin was obtained from Sigma-Aldrich (P4394; MO, USA) and reconstituted in normal saline at 1 mg/ml. Magnolol was obtained from Sigma-Aldrich (M3445) and reconstituted in DMSO at 10 mM.

### Cells

The murine Lewis lung carcinoma (LLC) cell line was obtained from American Type Culture Collection (CRL-1642; VA, USA) and murine colon carcinoma (CT-26) cell line was purchased from Korean Cell Line Bank (80009; Seoul, Korea). The cells were cultured with Dulbecco's modified Eagle's medium (LM001-05; Welgene, Daegu, Korea) supplemented with 10% heat-inactivated fetal bovine serum (S001-07; Welgene), 100 U/mL penicillin, and 100 μg/mL streptomycin (15140122; Invitrogen, CA, USA). The cells were maintained at 37°C in a humidified incubator containing 5% CO_2_ and cultured every 2–3 days until reaching 80% confluence.

### Animals

C57BL/6 wild-type mice (6-week-old, 20–22 g, male) were purchased from DBL (Chungcheongbuk-do, Korea). All animals were maintained in a pathogen-free environment on a 12-h light/dark cycle with free access to food and water. The animal studies were approved by the University of Kyung Hee Institutional Animal Care and Use of Committee (KHUASP(SE)-18-118). For the cisplatin-induced sarcopenia mouse model, 2.5 mg/kg cisplatin was administered daily for 5 days on days 1–5 and days 26–30 for a total of 10 times. We used maximal cisplatin dose with complete mice survival to avoid systemic injury by excessive toxicity following the previous investigation by Sawhney et al. (27). Mice received 1, 5, or 10 mg/kg magnolol every 3 days. All drugs were intraperitoneally injected. Body weight and food uptake were measured every 3 days during the experiments. After the termination of experiments, blood was drawn by cardiac puncture under anesthesia (2% isoflurane), and hind-leg muscles (tibialis anterior: TA, extensor digitalis longus: EDL, soleus: SOL) were harvested. All mice were euthanized by isoflurane and cervical dislocation. The representative images of hind legs were captured digitally using a SONY NEX-5 digital camera (SONY Corp., Tokyo, Japan), and muscle mass was measured by weight.

For the tumor-bearing mouse model, 5 × 10^4^ LLC cells with 50% Matrigel matrix (354234; Corning, NY, USA) were injected subcutaneously to the right flank per mouse. Three days after tumor inoculation, mice were received 10 mg/kg magnolol every 3 days a total of 5 times. Cisplatin (2.5 mg/kg) was injected daily for 5 days, beginning at day 7 after tumor inoculation.

### Renal Toxicity Analysis

Blood was incubated at room temperature for clotting for 3 h and centrifuged (3,000 rpm, 4°C, for 30 min). Supernatant was collected and blood urea nitrogen (BUN) and creatinine concentration was measured at Genia Inc. (Seongnam, Korea).

### Grip Test

All-limbs or forelimb grip strength were measured using a digital force gauge (DS2-5N; IMADA Inc., IL, USA). The gauge was placed horizontally for all-limbs tests and vertically for forelimb tests. As mice were placed on a grid for the all-limbs test or grasped a bar for the forelimb test, mice's tails were slowly pulled backwards or downwards 3–5 times to record the peak tension at the time that the mice released their paws (28). All tests were repeated thrice at 30 min intervals, and the average of 3 values was used for calculations. The values in which the mouse leaved the bar without resistance before pulling back or downwards were excluded.

### Proliferation Assay

For cancer cell proliferation assay *in vitro*, CT-26 and LLC cells were seeded in 96-well plate at a density of 1 × 10^3^ cells/well. The next day, cells were treated 0.1, 1, or 10 μM magnolol. After 24, 48, and 72 h exposure, cell proliferation was detected using CellTiter 96® AQueous One Solution Cell Proliferation Assay kit (Promega, WA, USA) following the manufacturer's instruction. For *in vivo* proliferation assay, 100 μl of 10 mg/ml bromodeoxyuridine (BrdU) solution (550891; BD bioscience, CA, USA) was intraperitoneally injected (1 mg per mouse) 3 h before sacrifice. BrdU-labeled cells were stained with anti-BrdU antibody and detected using flow cytometry.

### Flow Cytometry Analysis

Splenocytes were dissociated into single cells using a 40-μm nylon mesh strainer. Red blood cells were lysed with Pharmlyse buffer (555899; BD Bioscience), and single cells were stained for 1 h at 4°C using the following antibodies to observe CD4 T cells (CD45^+^CD4^+^CD8^−^), CD8 T cells (CD45^+^CD4^−^CD8^+^), total macrophages (CD45^+^CD11b^+^F4/80^+^): anti-mCD45-FITC (103108; BioLegend, clone: 30-F11), anti-mCD4 APC-e-Fluor 780 (47-0041-82; e-bioscience, clone: GK1.5), anti-mCD8 Percp-cy5.5 (45-0081-80; e-bioscience, clone: 53-6.7), anti-mCD11b BV510 (101245; BioLegend, clone: M1/70), and anti-mF4/80 BV421 (123131; BioLegend, clone: BM8). M1 (CD206^−^CD163^−^), M2a (CD206^+^CD163^−^), and M2c (CD206^+^CD163^+^) macrophages were classified within CD45^+^CD11b^+^F4/80^+^ macrophages by staining with the following antibodies: anti-mCD206 APC (141707; BioLegend, clone: C068C2) and anti-mCD163 PE (12-1631-82; e-bioscience, clone: TNKUPJ).

For muscle infiltrating cell analysis, TA muscle tissue was cut and incubated in 1 unit/ml DNase I and 2.5 mg/ml Liberase TL (5401020001; Roche, IN, USA) for 1 h at 37°C. The tissues were dissociated using gentleMACS™ Dissociator (Miltenyi Biotec, Bergisch Gladbach, Germany). Surface markers were stained for 30 min at 4°C using following antibodies: anti-mCD45 APC-e-flour 780 (47-0543-80; e-bioscience, clone: A20), anti-mCD11b Percp-cy5.5 (101227; BioLegend, clone: M1/70), anti-mF4/80 FITC (11-4081-81; e-bioscience, clone: BM8), anti-mCD163 PE (12-1631-82; e-bioscience, clone: TNKUPJ), and anti-mCD86 PE-cyanine7 (105013; BioLegend, clone: GL-1). After washing out, the cells were fixed and permeabilized for intracellular staining. For BrdU detection, anti-BrdU BV510 (563445; BD bioscience, clone: 3D4) was diluted in permeabilization buffer and incubated for 1 h. IGF-1 was indirectly stained by rabbit anti-IGF-1 (1:1,000; ab9572; Abcam) primary antibody and goat anti-rabbit IgG-Alexa Fluor 405 (1:1,000; A-31556; Invitrogen) secondary antibody for 1 h respectively.

The stained cells were detected on BD FACSLyric instruments (BD bioscience, CA, USA) after being washing and were analyzed using FlowJo software (Treestar Inc., CA, USA).

### Histological Analysis

TA muscle tissues were fixed for 24 h in 10% neutral buffered formalin. The tissues were dehydrated in 70, 80, 90, and 100% ethanol. After soaking in ethanol:xylene=1:1 and xylene, tissues were embedded in paraffin. The tissues were cut on a rotary microtome at 4-μm thickness, deparaffinized and rehydrated. H&E staining was performed, and all sections were imaged on an Olympus microscope (Tokyo, Japan). The TA fiber diameter on cross sections was analyzed in three random fields per section. The diameter was calculated using ImageJ software by converting the fiber area into diameter after measuring the area of the individual fibers in segmented image. Fibers on the edge of the images were excluded.

### Immunofluorescence Staining

TA muscle tissues sections were rehydrated and blocked with 1.5% BSA (in PBS) for 1 h at room temperature (RT). The sections were incubated overnight with mouse anti-mouse myosin heavy chain (1:1,000; MAB4470; R&D Systems, MN, USA) and rat anti-mouse CD68 (1:1,000; MCA1957GA; Bio-Rad, CA, USA) primary antibodies at 4°C and visualized with Alexa Fluor 488-conjugated goat anti-mouse IgG (A28175; Invitrogen, CA, USA) and Alexa Fluor 594-conjugated goat anti-rat IgG (A11007; Invitrogen) secondary antibodies for 1 h at RT to investigate the macrophage infiltration. The number of macrophages were counted by an observer blinded and expressed as count per area.

CD163 expression on CD68^+^ macrophages were detected with rabbit anti-mouse CD68 (1:1,000; Santa Cruz, CA, USA), rat anti-CD163 (1:1,000; 14-1631-82; e-bioscience, CA, USA), Alexa Fluor 488-conjugated goat anti-rabbit IgG (A11008; Invitrogen), and Alexa Fluor 594-conjugated goat anti-rat IgG (A11007; Invitrogen). For the detection of IGF-1 expression on CD68^+^ macrophages, the sections were incubated with rat anti-mouse CD68 (1:1,000; MCA1957GA; Bio-Rad) and rabbit anti-mouse IGF-1 (1:1,000; 102408; Abcam) primary antibodies. The markers were visualized with Alexa Fluor 488-conjugated goat anti-rat IgG (A11006; Invitrogen) and Alexa Fluor 594-conjugated goat anti-rabbit IgG (A32740; Invitrogen) secondary antibodies. All images were captured minimum 5 fields randomly per section and the maximum and minimum values were excluded from the analysis. The mean values of three different sections per mouse were used.

### IGF-1 Immunoassay

TA muscle was lysed with RIPA buffer supplemented with protease inhibitor cocktails by homogenization using mechanical homogenizer (Precellys® 24; Bertin, France). Enzyme-linked immunosorbent assay (ELISA) kits were purchased from R&D systems (DY791; MN, USA) and were used to determine the levels of IGF-1 in TA muscle following manufacturer's instruction. The absorbance was read at 450 nm.

### Differentiation of Bone Marrow-Derived Macrophages (BMDMs)

Bone marrow cells were flushed out from the femurs of C57BL/6 mice into PBS. Red blood cells were lysed, and cells were resuspended in RPMI1640 supplemented with 10 ng/ml macrophage colony-stimulating factor (M-CSF; 416-ML; R&D systems). The cells were seeded at 2 × 10^6^/ml density and cultured for 7 days to differentiate into M0 macrophages. The differentiation medium was replaced every 3 days. After 7 days, the cells were replated in 6-well plate (1 × 10^6^/well) and exposed to M-CSF containing medium with 10 ng/ml LPS (L4391; Sigma-Aldrich) or 20 ng/ml murine recombinant IL-4 (404-ML; R&D systems) for 24 h to induce M1 or M2 macrophages. At the same time, cells were treated with 0.1, 1, or 10 μM to investigate the effect of magnolol on macrophage phenotypes.

### Real-Time Quantitative PCR

Total RNA from cultured BMDMs or TA muscles was extracted with easy-BLUE™ (17061; iNtRON, Sungnam, Korea). cDNA synthesis was performed using CycleScript reverse transcriptase (BIONEER, Daejeon, Korea), following the manufacturer's instruction. Expression levels were measured by real-time PCR amplification using SYBR Green. Signals are expressed using the standard 2^−ddCt^ method after normalizing to the reference signal glyceraldehyde-3-phosphate dehydrogenase (GAPDH). The following primers were used: *Gapdh* (forward: ACC CAG AAG ACT GTG GAT GG, reverse: CAC ATT GGG GGT AGG AAC AC), *Nos2* (forward: GGC AGC CTG TGA GAC CTT TG, reverse: CAT TGG AAG TGA AGC GTT TCG), *Cd163* (forward: AGGCCACACCTCCTAAACCT, reverse: TCTGCCATCTGCTTTCATTG), *Mmp8* (forward: CTTGCACTCTCGATGGACAA, reverse: TTGCACAGACACATTGCTGA), and *Igf1* (forward: CGATACTCGCTCTGTGTCCA, reverse: GTTGGTTTGTGGGTTCTGCT).

### Statistical Analysis

Parameters are expressed as the mean ± standard error of the mean (SEM). The comparisons were conducted using one-way ANOVA followed by Turkey's test or two-way ANOVA based on the two-way Bonferroni post-test for multiple comparisons by Prism 5.01 software (GraphPad Software, Inc.). Unpaired *t*-test was used for the comparison of two independent groups. *P* < 0.05 was considered significant.

## Results

### Amelioration of Body Weight Loss and Renal Dysfunction by Magnolol Treatment

**Figure 1:**
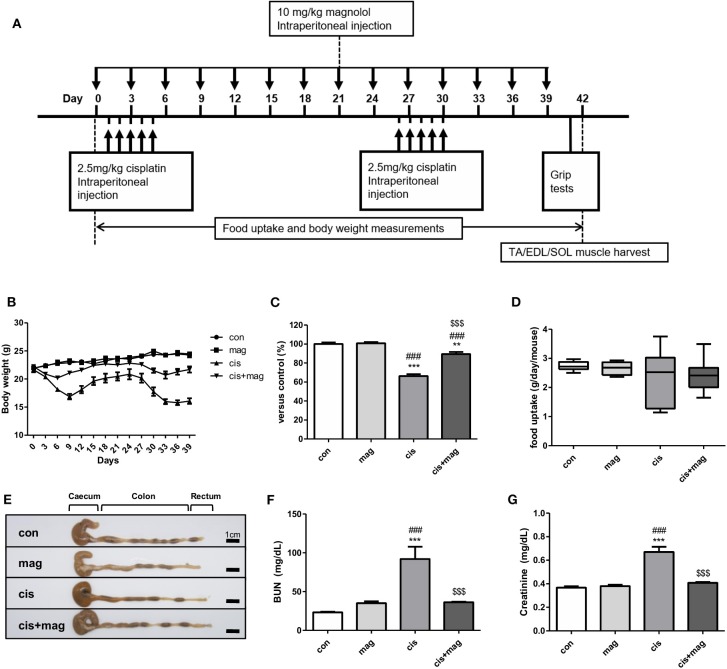
Magnolol prevented cisplatin-induced body weight loss and systemic dysfunction. **(A)** Experimental schedule of cisplatin and magnolol injection. Briefly, muscle wasting was induced by cisplatin injection (2.5 mg/kg daily, total 10 times) and the protective effect of magnolol treatment (10 mg/kg, every 3 days) was observed. All mice were sacrificed on day 42. **(B)** Changes in body weight in a cisplatin-induced muscle wasting mouse model. **(C)** Relative body weight changes of control (con), magnolol (mag), cisplatin (cis), or both cisplatin and magnolol treated mice (cis+mag) were expressed as% vs. control (day 42). **(D)** Mean daily food uptake per mouse during whole period, and **(E)** representative images showing caecum, colon, and rectum (Scale bar: 1 cm). **(F)** Concentration of blood urea nitrogen (BUN) and **(G)** creatinine in blood serum after the termination of the experiment (day 42). All results are representative from three individual experiments (*n* = 6–7), and the graphs are expressed as the mean ± SEM. **P < 0.01; ***P < 0.001 vs. con, ^*###*^*P* < 0.001 vs. mag, and ^$$$^*P* < 0.001 vs. cis based on the one-way ANOVA Tukey's test. The letters for no significance were not shown.

We initially tested the protective effect of magnolol using a sarcopenia mouse model. Cisplatin is a standard chemotherapeutic agent and induces rapid muscle loss. We thus induced the muscle wasting via cisplatin injection once daily on days 1–5 and days 26–30 at 2.5 mg/kg (total 25 mg/kg) (Figure 1A). Magnolol (10 mg/kg) was administered every 3 days for 40 days. Figure 1A shows the experimental schedule that was employed to induce loss of weight and muscle for long term observation with complete mouse survival and less systemic toxicity (27). Cisplatin caused a marked decrease of body weight compared with control. After the initial five injections of cisplatin, the body weight was rapidly decreased and recovered after day 9. However, after the second period of cisplatin injection, body weight recovery failed (Figure 1B). On the other hand, magnolol administration to cisplatin-injected mice significantly protected against body weight loss, and mice recovered after days 6 and 33. Magnolol alone caused no significant change in body weight (Figures 1B,C). We measured the mean daily food uptake per mouse during the entire experiment to verify whether cisplatin or magnolol induces anorexia, but there was no significant difference among all groups (Figure 1D). Neither cisplatin nor magnolol changed the colon length, which is associated with intestinal damage during chemotherapy (Figure 1E). As renal failure accompanied with systemic inflammation is prominently associated with skeletal muscle breakdown, we also checked the concentration of BUN and creatinine to verify the effect of magnolol on cisplatin-induced renal damage. Magnolol alone did not change the concentration of BUN and creatinine. Both BUN and creatinine were significantly higher in cisplatin-treated mice compared to control mice whereas magnolol treatment ameliorated these changes (Figures 1F,G). No death was recorded in all groups.

### Protective Effect of Magnolol on Cisplatin-Induced Muscle Wasting

**Figure 2:**
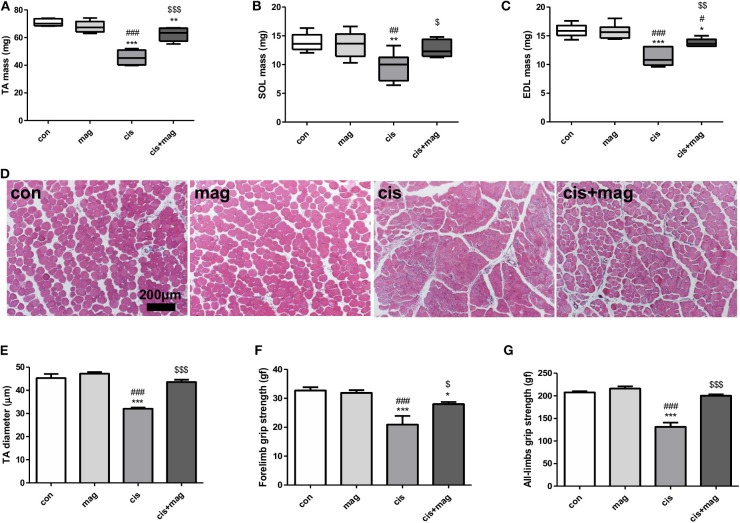
Magnolol protected cisplatin-induced muscle wasting. Protective effect of magnolol on muscle was assessed by measuring muscle mass, fiber diameter, and grip strength in the cisplatin-induced muscle wasting mouse model. Hind leg muscle tissues were harvested from control (con), magnolol (mag), cisplatin (cis), or cis+mag mice at the end of the experiment (day 42). **(A–C)** Muscle mass of **(A)** tibialis anterior (TA), **(B)** extensor digitalis longus (EDL), and **(C)** soleus (SOL). **(D)** Histological images of hematoxylin and eosin staining of TA muscle tissue (scale bar: 200 μm) and **(E)** measurements of fiber diameter in transverse sections. The grip strength of **(F)** forelimbs and **(G)** all-limbs measured using a digital force gauge (day 41). All data are representative of three individual experiments (*n* = 7), and presented as the mean ± SEM. *P < 0.05; **P < 0.01; ***P < 0.001 vs. con, ^#^*P* < 0.05; ^*##*^*P* < 0.01; ^*###*^*P* < 0.001 vs. mag, and ^$^*P* < 0.05; ^$$^*P* < 0.01; ^$$$^*P* < 0.001 vs. cis based on the one-way ANOVA Tukey's test. The letters for no significance were not shown.

Next, we assessed the effect of magnolol on skeletal muscle. Measurement of the muscle weight of TA, EDL, and SOL indicated that magnolol protected against muscle loss induced by cisplatin injection (Figures 2A–C). Cisplatin significantly decreased the myofiber diameter, while magnolol markedly prevented this change. Magnolol alone did not change the TA muscle cross-sectional diameter (Figures 2D,E). Further, cisplatin administration significantly decreased all-limbs and forelimb grip strength, whereas magnolol treatment prevented these changes (Figures 2F,G).

To further confirm the optimal dose of magnolol treatment for the prevention of muscle wasting, we used different concentrations of magnolol of (1, 5, and 10) mg/kg with the same experimental schedule as shown in Figure 1A. As a result, (1, 5, and 10) mg/kg of magnolol successfully prevented body weight loss induced by cisplatin. Different concentrations of magnolol alone did not induce body weight changes (Supplementary Figures 1A,B). Unexpectedly, the protective effect of magnolol on grip strength was not dose-dependent and was inversely related to the dosage (Supplementary Figure 1C). In addition, grip strength was significantly increased in the 1 mg/kg magnolol-treated group compared with the healthy control. Measurement of muscle mass also showed a similar tendency. In addition, (1, 5, and 10) mg/kg magnolol treatment protected the loss of TA, EDL, and SOL muscle by cisplatin injection, and 1 mg/kg magnolol showed the best protective effect (Supplementary Figures 1D–G). TA muscle fibers were damaged and tapered in the cisplatin group compared with control, whereas the fiber damage was significantly protected in all magnolol groups cotreated with cisplatin. Different concentrations of magnolol alone did not change myofiber thickness (Supplementary Figures 1H,I).

### Increase of IGF-1 Level and Macrophage Infiltration in Skeletal Muscle by Magnolol Treatment

**Figure 3:**
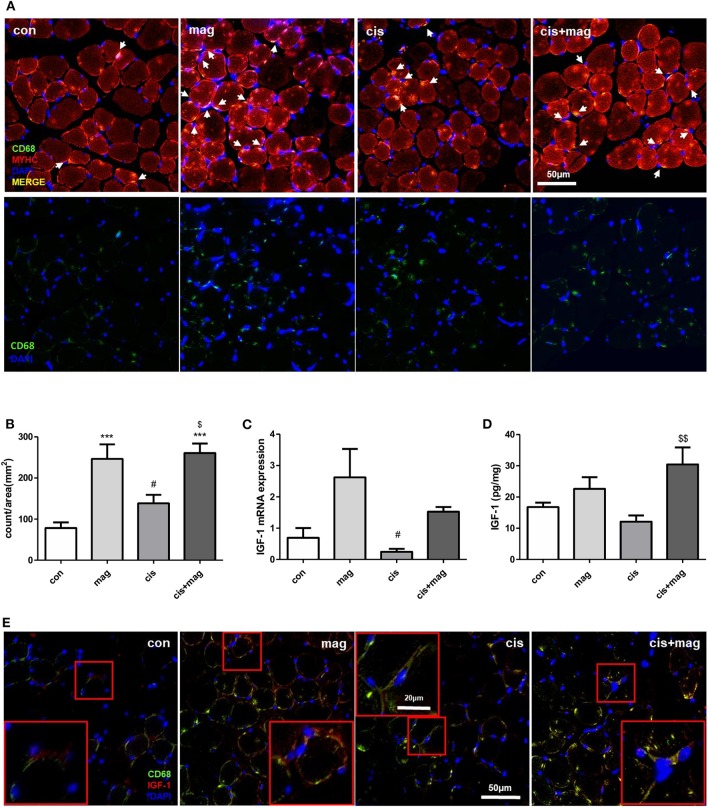
Magnolol increased macrophages and IGF-1 expression in TA muscle. Cross-sections and total mRNA or protein were obtained from TA muscles in the control (con), magnolol (mag), cisplatin (cis), or cis+mag groups. **(A)** Immunofluorescence staining for anti-myosin heavy chain (MYHC; red) and CD68 macrophages (green) in TA muscle tissues (merged yellow pixels: upper panel, green channel only: lower panel). 4'6-Diamidino-2-phenylindole (DAPI) was used to stain the nuclei (blue). White arrows indicate the CD68-stained cells near DAPI. Magnification: ×40, Scale bar: 50 μm. **(B)** Count of CD68^+^ macrophages (white arrows in **A**) per image area (mm^2^). At least 5 random fields per section were analyzed. The events that do not contain DAPI staining were not counted. **(C)** IGF-1 mRNA expression measured by qPCR in TA muscle tissues. GAPDH was used as reference and the level of the genes was normalized to 1. **(D)** Quantification of IGF-1 protein expression by ELISA in TA muscle tissues. **(E)** Immunofluorescence staining showing IGF-1 (red) and CD68^+^ macrophages (green) in TA muscle. Magnification: ×40, Scale bar: 50 μm. CD68^+^ macrophages with IGF-1 expression were enlarged in red box. Magnification: ×40, Scale bar: 20 μm. Data are representative of three individual experiments, and all graphs are expressed as the mean ± SEM of 5 mice. ***P < 0.001 vs. con, ^#^*P* < 0.05 vs. mag, and ^$^*P* < 0.05; ^$$^*P* < 0.01 vs. cis based on the one-way ANOVA Tukey's test. The letters for no significance were not shown.

After skeletal muscle damage, leukocytes such as neutrophils and macrophages quickly infiltrate into the injured site and regulate muscle stem cell activation during regeneration (29). The absence of macrophages, which are an important source of chemokines, matrix metalloproteinases (MMPs), and other mediators supporting tissue remodeling in muscle injury models, resulted in the failure of muscle protection (30). Thus, we investigated whether the protective effect of magnolol is associated with the macrophages in skeletal muscle. The macrophages infiltrating TA muscle was detected by CD68 immunofluorescence staining, and myosin heavy chain and diamidino-2-phenylindole (DAPI) were used as counterstains. Cisplatin injection induced CD68^+^ macrophage accumulation after injury compared with control (Figure 3A). Importantly, magnolol significantly increased CD68^+^ macrophage infiltration into TA muscle regardless of cisplatin administration (Figures 3A,B). We further confirmed the IGF-1 expression, which is associated with myogenesis and protein synthesis (31). The IGF-1 mRNA levels were higher in the magnolol and cis+mag group than in the control group, however, there were no statistically significant differences. Notably, IGF-1 protein production was significantly increased in the cis+mag group compared to the cisplatin group in TA muscle, although no significant difference was observed between the magnolol group and the control group (Figures 3C,D). Immunostaining of CD68 and IGF-1 in TA muscle tissue showed that macrophages express IGF-1 (Figure 3E).

**Figure 4:**
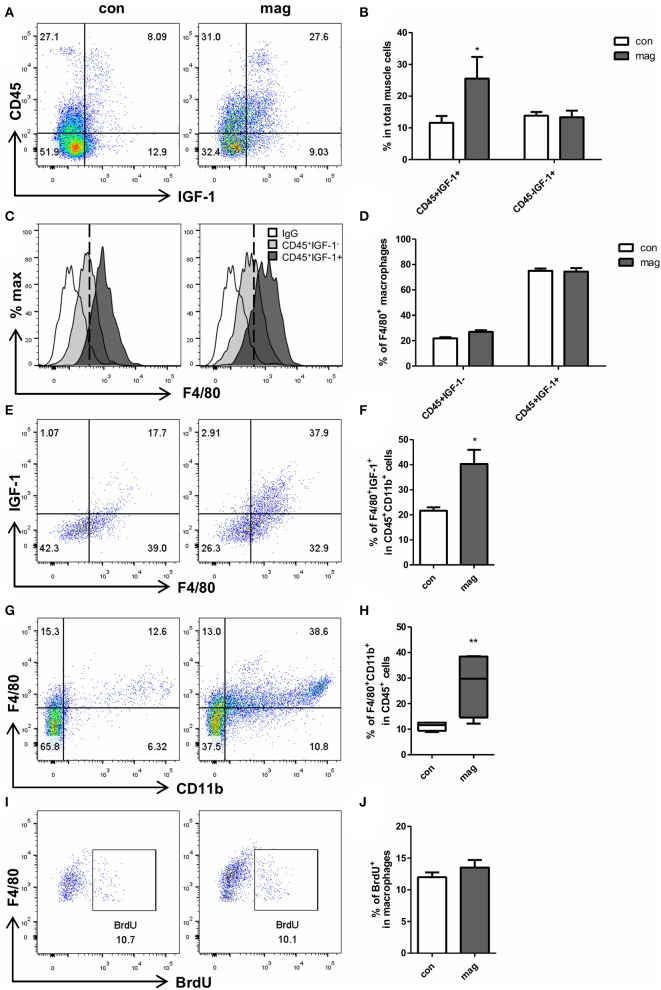
Magnolol increased the infiltration of IGF-1^+^ macrophages into muscle tissue. Wild type mice were given vehicle (con) or 10 mg/kg magnolol (mag) intraperitoneally 3 times for a week. Single cells were isolated from TA muscle and analyzed using flow cytometry. BrdU was injected 3 h before sacrifice for proliferation assay. **(A)** Representative dot plots of CD45 vs. IGF-1 within total single cells from TA muscle tissues. **(B)** Bar graph shows the percentage of CD45^+^IGF-1^+^ and CD45^−^ IGF-1^+^ populations in total cells. **(C)** Histograms showing the cell counts based on the F4/80 expression (white: isotype control; filled light gray: gated on CD45^+^IGF-1^−^; filled dark gray: gated on CD45^+^IGF-1^+^). **(D)** Percentages of F4/80^+^ macrophages in IGF-1^+^ or IGF-1^−^ cells among the CD45^+^ immune cells. **(E,F)** Percentages of IGF-1 expressing F4/80^+^ macrophages measured by gating on CD45^+^CD11b^+^ cells. **(G)** Representative FACS plots of CD11b^+^F4/80^+^ macrophages gated on CD45^+^ cells in muscle tissue and **(H)** bar graph showing the percentage of CD11b^+^F4/80^+^ cells in CD45^+^ cells. **(I,J)** Frequency of BrdU^+^ proliferating populations in CD11b^+^F4/80^+^ macrophages. All graphs are presented as the mean ± SEM (*n* = 4). *P < 0.05; **P < 0.01 vs. con based on the unpaired t test. The letters for no significance were not shown.

To ensure the effect of magnolol on macrophages, we additionally tested whether the short-term injection of magnolol (total 3 injections, 10 mg/kg) increases the number of IGF-1^+^ macrophages in TA muscles using flow cytometry. Gating strategies are shown in Supplementary Figure 2. We confirmed that magnolol treatment significantly increased the IGF-1^+^CD45^+^ cells, but the IGF-1 expression did not differ within CD45^−^ myogenic cells (Figures 4A,B). Both in control and magnolol mice, the majority of the IGF-1^+^ immune cells were F4/80^+^ macrophages (Figures 4C,D). Moreover, the percentage of IGF-1^+^ F4/80^+^ macrophages in CD45^+^CD11b^+^ cells was significantly higher in the magnolol group compared to the control group (Figures 4E,F). We further confirmed that magnolol significantly increased the number of macrophages within CD45^+^ leukocytes in TA muscle compared to control (Figures 4G,H). To determine whether the increase in macrophages is due to infiltration or proliferation, we compared the BrdU^+^ populations in CD11b^+^F4/80^+^ macrophages. The percentage of macrophages that had incorporated BrdU did not differ between control and magnolol groups, indicating that magnolol increased the infiltration of macrophages without affecting proliferative activity (Figures 4I,J). In aggregation, the data suggested that magnolol treatment increased the infiltration of macrophages, one of the major sources of IGF-1 in muscle tissues.

### Changes of Macrophage Subtypes Induced by Magnolol Treatment

**Figure 5:**
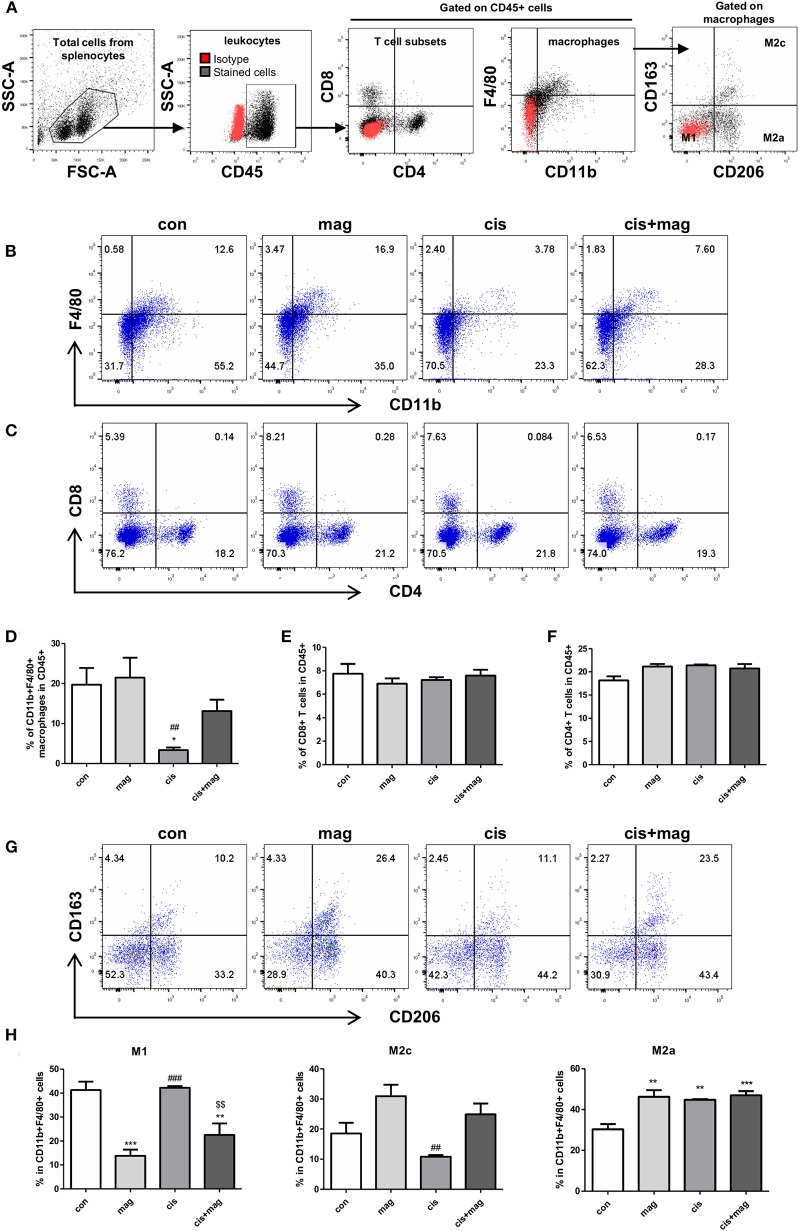
Magnolol increased M2c-like macrophage subpopulations. Changes in immune cell subtypes by magnolol treatment were determined using flow cytometry in splenocytes from either control (con), magnolol (mag), cisplatin (cis), or cis+mag mice. **(A)** Gating strategy for analysis of macrophages and T cells subsets. Red dots display isotype controls and black dots show the stained cells with specific antibodies. **(B)** Representative dot plots of CD11b^+^F4/80^+^ macrophages, **(C)** CD8^+^ and CD4^+^ T cells. **(D)** Bar graphs showing mean% of CD11b^+^F4/80^+^ macrophages, **(E)** CD8^+^ T cells, and **(F)** CD4^+^ T cells in CD45^+^ total leukocytes in splenocytes. **(G,H)** Analysis of macrophage subtypes. **(G)** Representative dot plots identifying macrophages as CD163^−^CD206^−^ M1, CD163^−^CD206^+^ M2a, and CD163^+^CD206^+^ M2c gated on CD45^+^CD11b^+^F4/80^+^ cells. **(H)** Bar graphs showing mean% of M1, M2a, and M2c macrophages in CD11b^+^F4/80^+^ splenocytes. All data are presented as the mean ± SEM (*n* = 5). *P < 0.05; **P < 0.01; ***P < 0.001 vs. con, ^*##*^*P* < 0.01; ^*###*^*P* < 0.001 vs. mag, and ^$$^*P* < 0.01 vs. cis based on the one-way ANOVA Tukey's test. The letters for no significance were not shown.

We further investigated whether the magnolol treatment affected the different immune cells using flow cytometry analysis (gating strategies are shown in Figure 5A). In spleen, the percentage of CD11b^+^F4/80^+^ macrophages in CD45^+^ leukocytes was significantly decreased by cisplatin administration compared to control (Figures 5B,D). However, magnolol treatment prevented the loss of macrophages induced by cisplatin. Magnolol alone did not alter the number of macrophages. We then verified the change in T cell subsets (Figure 5C), but no differences in CD8 and CD4 T cells were noted in all groups (Figures 5E,F). Next, the changes in macrophages subsets were investigated by defining CD163^−^CD206^−^ cells as M1, CD163^−^CD206^+^ cells as M2a, and CD163^+^CD206^+^ cells as M2c phenotype gated on CD11b^+^F4/80^+^ macrophages in CD45^+^ cells (Figure 5G). M1 macrophages were significantly decreased upon magnolol treatment compared to control. Cisplatin did not change the percentage of M1 macrophages. Cis+mag groups showed significantly lower M1 levels compared to the cis group. Importantly, M2c macrophages showed a converted tendency with M1 macrophages, but there was no significance difference between the cis and cis+mag groups. Although magnolol also increased the number of M2a macrophages compared to control, there was no difference in cisplatin-treated groups (Figure 5H).

**Figure 6:**
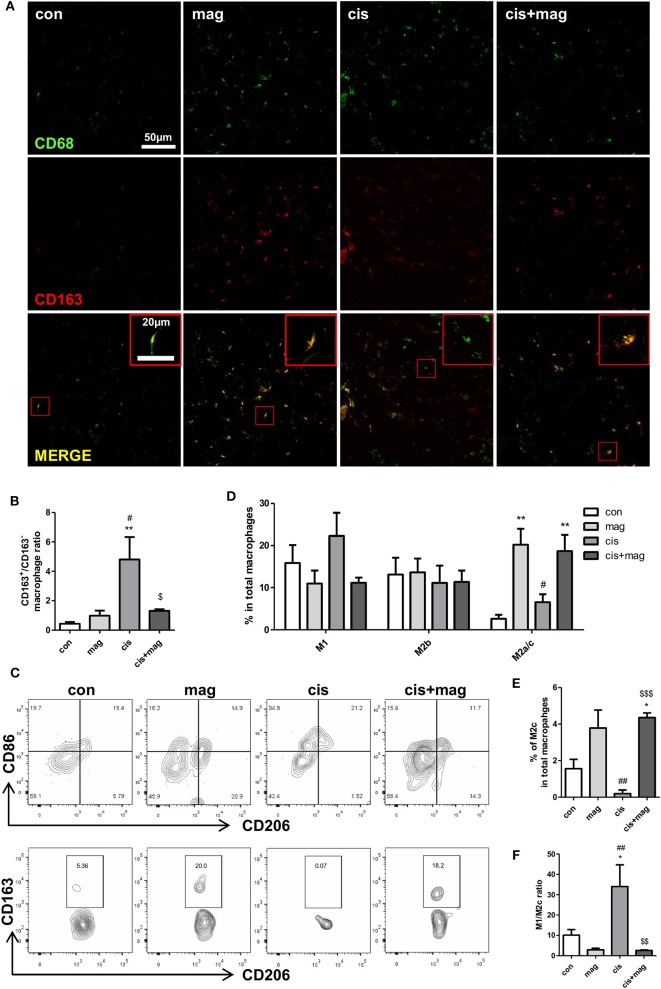
Magnolol attenuated the M1/M2 imbalance caused by cisplatin damage. Phenotypic changes in macrophages by magnolol treatment were analyzed in TA muscle tissue using immunostaining and flow cytometry. TA muscles were obtained from control (con), magnolol (mag) cisplatin (cis), or cis+mag mice at the end of the experiment (day 42). **(A)** Macrophage phenotyping using immunofluorescence staining with the pan-macrophage marker CD68 (green) and M2 regenerative macrophage specific marker CD163 (red). Magnification: ×40, Scale bar: 50 μm. Merged macrophages were enlarged in red box, Scale bar: 20 μm. **(B)** CD163^+^/CD163^−^ ratio calculated by dividing the numbers of CD68^+^CD163^+^ merged yellow pixel by those of CD68^+^CD163^−^ green pixel. **(C)** Flow cytometry analysis of CD86^+^CD206^−^ M1, CD86^+^CD206^+^ M2b, CD86-CD206^+^CD163^−^ M2a, and CD86^−^CD206^+^CD163^+^ M2c macrophages. Representative contour plots of macrophages characterized by CD86 and CD206 gated on CD45^+^CD11b^+^F4/80^+^ subsets (upper panel) and M2a or M2c macrophages characterized by CD163 and CD206 gated on CD86^−^CD206^+^ subsets (lower panel). **(D)** Bar graphs showing mean% of M1, M2b, and M2a/c macrophages in CD11b^+^F4/80^+^cells and **(E)** mean% of M2c macrophages in CD11b^+^F4/80^+^cells. **(F)** M1/M2c ratio calculated based on the number of M1 and M2c macrophages in CD45^+^CD11b^+^F4/80^+^ gated cells. Data represent mean ± SEM of 5 mice, and all data are representative from three individual experiments. *P < 0.05; **P < 0.01 compared to con, ^#^*P* < 0.05; *P < 0.05 compared to mag, and ^$^*P* < 0.05; ^$$^*P* < 0.01; ^$$$^*P* < 0.001 compared to cis using Turkey's test. The letters for no significance were not shown.

We next asked whether magnolol induced the change of macrophage subtypes infiltrated in TA muscle tissue. Infiltrated macrophages were stained with CD68 and CD163^+^/CD163^−^ ratio was calculated based on the pixels of yellow merged population or CD68 single positive population. The CD163^+^/CD163^−^ ratio in the cisplatin group was significantly higher compared to control, and magnolol treatment effectively blocked the increase in the ratio (Figures 6A,B). We further analyzed four distinct subpopulations in CD45^+^CD11b^+^F4/80^+^ macrophages in muscle by flow cytometry: CD86^+^CD206^−^ M1, CD86^+^CD206^+^ M2b, and CD86^−^CD206^+^ M2a/M2c macrophages (Figures 6C,D). Inflammatory M1 macrophages were slightly increased by cisplatin treatment (no significance), however, magnolol inhibited the increase of M1 macrophages. At the same time, CD86^−^CD206^+^ M2a/M2c macrophages were significantly increased by magnolol treatment. There was no change in M2b populations. Additionally, we verified that the percentage of CD86^−^CD206^+^CD163^+^ M2c macrophages in total macrophages was significantly increased by magnolol (Figure 6E). These changes ameliorated the M1/M2c imbalance induced by cisplatin (Figure 6F).

### Effect of Magnolol on Bone Marrow-Derived Macrophage Activation *in vitro*

**Figure 7:**
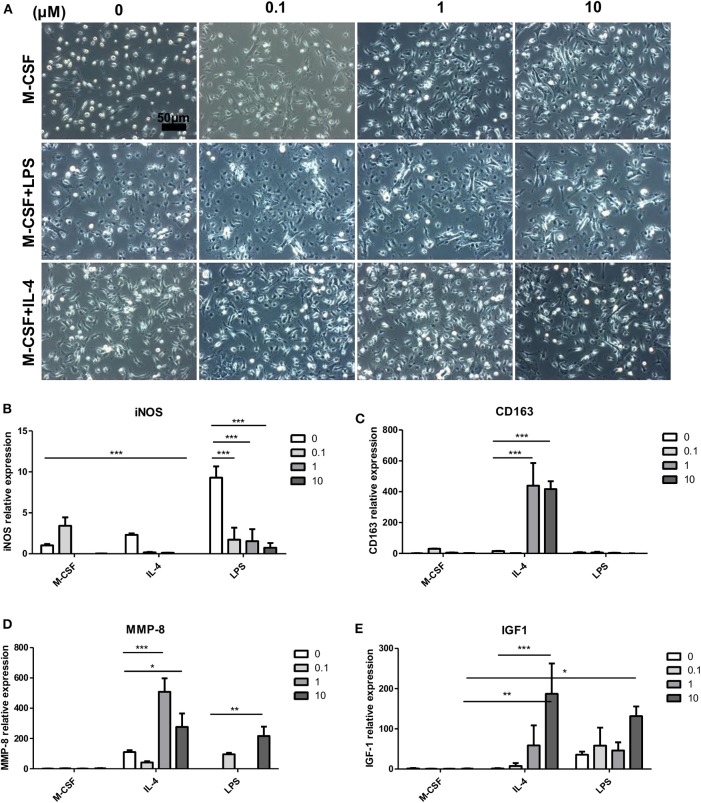
Magnolol modulated the activation of bone marrow-derived macrophages *in vitro*. To assess the effect of magnolol on macrophage activation, C57 bone marrow cells were differentiated into M0 (M-CSF), M1 (M-CSF+LPS), or M2 (M-CSF+IL-4) macrophages and treated with magnolol. **(A)** Images showing the cell morphology of M0, M1, or M2 macrophages treated with (0, 0.1, 1, and 10) μM magnolol (Scale bar: 50 μm). **(B–E)** mRNA quantification of **(B)** iNOS, **(C)** CD163, **(D)** MMP-8, and **(E)** IGF-1 as assessed by qPCR. The images are representative of three independent experiments, and the quantification results were calculated by averaging across three separate experiments. *P < 0.05; **P < 0.01; ***P < 0.001 using two-way Bonferroni post-test.

To determine whether magnolol directly alters macrophage phenotypes, we stimulated bone marrow cells with M-CSF for 7 days, and cells were cotreated magnolol with IL-4 for classical M2a or LPS for M1 polarization for 24 h on the last day. Cell morphology was observed after the stimulation (Figure 7A). Most of the cells in the M-CSF group without magnolol addition, which are categorized as M0-macrophages, exhibited a small round shape. However, magnolol-treated M0-macrophages exhibited more spindle-like M2 shapes. LPS-treated M1 macrophages exhibited flat-round/fried-egg shapes, but magnolol treatment resulted in a more spindle-like morphology. Magnolol did not result in marked changes in the spindle-like morphology within IL-4 M2a-polarized macrophages. Further, phenotypic changes were also observed based on gene expression. The M1-specific gene iNOS expression was increased after LPS treatment and was significantly suppressed after magnolol treatment (Figure 7B). M2c polarization-specific markers, CD163 and MMP-8 (32) were significantly increased after magnolol treatment in the IL-4-treated M2a groups. MMP-8 was also significantly increased in the LPS-treated M1 group upon magnolol treatment (Figures 7C,D). Plus, IGF-1 was significantly elevated with magnolol treatment both in the IL-4 and LPS groups (Figure 7E).

### Effect of Magnolol on Tumor Growth and Anti-cancer Activity of Cisplatin

**Figure 8:**
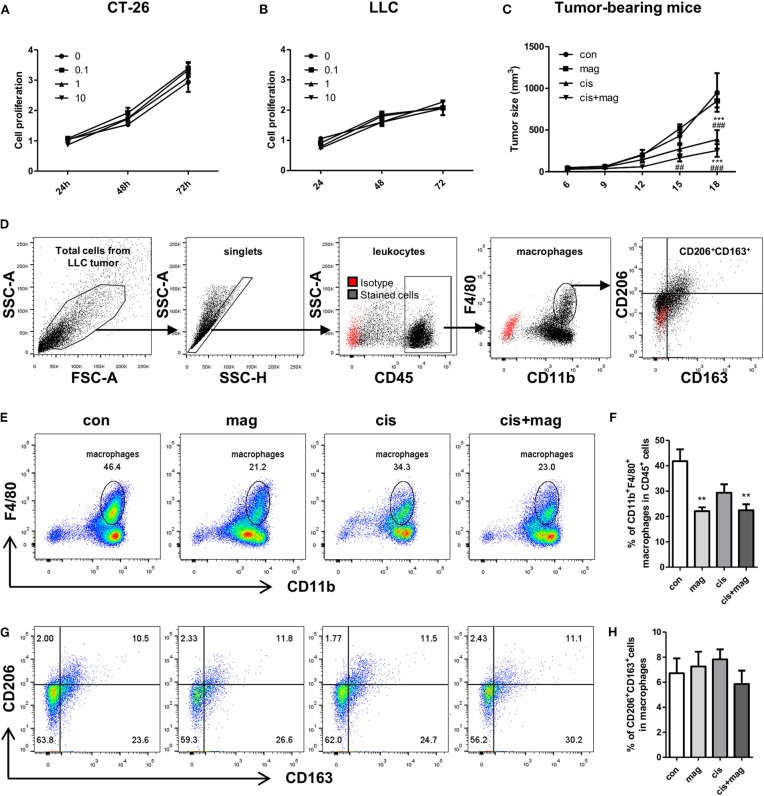
Magnolol did not increase tumor progression *in vitro* and *in vivo*. Effect of magnolol on tumor cell proliferation was determined using the MTS assay *in vitro*. **(A)** CT-26 and **(B)** LLC tumor cell proliferation after magnolol treatment at different concentrations (0, 0.1, 1, and 10 μM). **(C)** For *in vivo* assay, LLC tumor-bearing mice were administered vehicle (con), magnolol (mag), cisplatin (cis), or both (cis+mag). The graph shows mean tumor volume (mm^3^) measured using caliper. ***P < 0.001 vs. con and ^*##*^*P* < 0.01; ^*###*^*P* < 0.001 vs. mag based on the two-way Bonferroni post-test at a different time point. **(D–H)** Tumor tissues were dissociated into single cells and tumor-infltrated immune cells were analyzed by flow cytometry. **(D)** Gating strategy for analysis of tumor-infiltrated macrophages. Red dots show isotype controls and black dots indicate the stained cells with specific antibodies. **(E)** Representative FACS plots of CD11b vs. F4/80 and **(F)** bar graph showing the percentages of CD11b^+^F4/80^+^ macrophages in CD45^+^ cells. **(G)** Representative dot plots of CD163^+^CD206^+^ M2c macrophages gated on CD11b^+^F4/80^+^ cells and **(H)** mean percentages of M2c macrophages in tumor tissue. All graphs are expressed as the mean ± SEM of 5 mice. **P < 0.01 vs. con based on the one-way ANOVA using Tukey's test. The letters for no significance were not shown.

As magnolol increased the IGF-1 in macrophages, we next asked whether magnolol contributes to tumor growth and progression by recruiting M2-like macrophages into tumor microenvironment. We firstly tested the direct effect of magnolol on CT-26 and LLC lung cancer cell proliferation *in vitro* in three different concentrations (0.1, 1, and 10 μM). Tumor cells were incubated with vehicle or magnolol for 24, 48, and 72 h. Magnolol did not increase neither CT26 nor LLC tumor proliferation *in vitro* (Figures 8A,B). Next, we tested whether magnolol inhibits the anti-cancer activity of cisplatin in the tumor bearing mouse model. Magnolol alone did not change the tumor size compared to control. Cisplatin treatment significantly decreased the tumor growth and there was no difference in tumor growth between the cis and cis+mag groups (Figure 8C).

We next asked whether magnolol increases the infiltration of tumor-associated macrophages. Unexpectedly, both mag and cis+mag groups showed significant decrease in the percentages of infiltrated tumor-associated macrophages compared to the con group, whereas there was no significant difference between the con and cis groups (Figures 8D–F). As CD206^+^ and CD163^+^ M2-like tumor-associated macrophages have been associated with tumor progression and poor clinical prognosis (33–35), we also verified whether magnolol increases M2-like tumor associated macrophages. We quantitated the percentages of CD206^+^CD163^+^ M2-like subtypes in CD11b^+^F4/80^+^ tumor-associated macrophages and there was no significant difference among all groups (Figures 8G,H). Together, magnolol did not affect tumor progression and anti-cancer activity of cisplatin.

## Discussion

Our findings showed that magnolol treatment increased the infiltration of macrophages into muscle tissue. We further showed that increase of CD163^+^ M2c macrophages and IGF-1 expression by magnolol treatment attenuated the muscle atrophy in mice. *In vitro* studies also verified that magnolol promotes the differentiation of CD163^+^ M2 macrophages and IGF-1 expression. The cisplatin group showed a higher ratio of pro-inflammatory M1 macrophages in TA muscle, whereas magnolol treatment ameliorated the M1/M2c balance. Collectively, these results showed that magnolol attenuates the cisplatin-induced muscle wasting via increase of infiltration and activation of CD163^+^ M2c macrophages. These findings also emphasize the significant role of macrophages on muscle protection.

The previous study on anti-atrophic effect of magnolol by Chen et al. (24) also showed the decrease of inflammatory signals such as TNF-α, IL-6, and IL-1β in serum and whole muscle tissue after magnolol treatment. In this study, we hypothesized that the anti-inflammatory effect of magnolol is associated with immune cell infiltration, so that we focused on the role of magnolol on immune cell, not on the muscle fiber responses. Thus, we used 10 mg/kg magnolol following the study by Chen et al. As a result, we newly found that magnolol increased the magnolol infiltration and orchestrates M1/M2c macrophage balance in the cisplatin-induced muscle atrophy mouse model. Treatment of magnolol alone in normal mouse also increased the infiltration of M2c macrophages, but no treatment-related toxicity or abnormalities were observed. Moreover, dose dependent study at 1, 5, and 10 mg/kg revealed that 1 mg/kg magnolol, the lowest dose in this study, effectively ameliorated the muscle atrophy in cisplatin-injected mice and even improved the grip strength without an adverse effect in normal mice. These results suggest that magnolol treatment reached its maximal therapeutic levels below 1 mg/kg in the cisplatin-induced injury mouse model although the dose-dependent immune response is further needed to be performed.

Macrophages have highly heterogeneous phenotypes and can be additionally recruited or rapidly switch their types in response to the microenvironment or diseases. Macrophages often resemble each other and are difficult to precisely distinguish. They are mainly divided into two groups: classically activated M1 and alternatively activated M2 macrophages. Several studies have explored the inhibitory property of magnolol on RAW264.7 macrophage activation (25, 36), but the effect of magnolol on M2 macrophages remains unknown. Magnolol inhibits M1 activation related to NF-κB/Rel by blocking p38 kinase in RAW264.7 macrophages (25). NF-κB is a key regulator of the dynamic differentiation of macrophages. LPS stimulation of macrophages mediates STAT1, NF-κB, and phosphoinositide 3-kinase (PI3K) pathway activation. In addition, p50 NF-κB plays a divergent transcriptional role by promoting Pol II recruitment on M2 promoter genes (e.g., *ccl17* and *Arginase I*) and limiting its recruitment by M1 promoter genes (*Nos2, Ifn*-β, and *Tnf*-α) (37). Given that M2-related markers such as CD163 and MMP-8 were markedly increased in classically differentiated M2a BMDMs by magnolol treatment (Figure 7), magnolol may directly orchestrate the conversion of macrophage subtypes within M2 differentiated macrophages. Although additional studies are required to fully characterize the phenotypic changes of macrophages by magnolol treatment, we expect the process to be related to NF-κB regulation.

M2 phenotypes are expanded to M2a, M2b, and M2c based on the stimuli and transcriptional status (8, 38). M2a macrophages are induced by IL-4 and IL-13 stimulation and produce high levels of mannose receptor (CD206; *Mmr*). M2b macrophages, which are known as regulatory macrophages, are polarized by immune complexes and express high levels of chemokine (C-C motif) ligand 1, TNF-α, IL-1β, and IL-6, which result in the secretion of anti-inflammatory IL-10. M2c subtypes are induced by IL-10 and related to strong immunosuppression and tissue remodeling by secreting high levels of IL-10 and TGF-β. M2c macrophages present high levels of CD206, CD163, MMP-8, TIMP1, and MARCO, which are associated with angiogenesis and matrix remodeling (32). Especially, CD163 is considered as a M2c specific marker expressed predominantly on the cells (39, 40). Human M2c macrophages also express CD163 and high levels of CD206 (41). Several studies have been reported the M1 macrophages as CD206-negative cells whereas CD206 has been used for the classical marker of M2 macrophages (39, 42, 43). Following these lines of evidence, we defined the CD206^−^CD163^−^ macrophages as M1, CD206^+^CD163^−^ macrophages as M2a, and CD206^+^CD163^+^ macrophages as M2c subtype in this study. In Figure 6A, we showed the changes of macrophage subtypes by magnolol treatment using CD68/CD163 immunostaining. However, these markers are not specific to M2c macrophages since activated monocytes and dendritic cells can also express CD68 (44, 45). In addition, most of the macrophages in cisplatin-injected mice were CD68^+^CD163^−^ that contain both M1 and M2b subpopulations. For these reasons, we also performed flow cytometry analysis for the phenotypic characterization of macrophages in skeletal muscle using the antibodies against several surface markers such as CD45, CD11b, F4/80, CD86, CD206, and CD163 (Figure 6C) and found that magnolol treatment induces the increase of CD86^−^CD206^+^CD163^+^ M2c macrophages.

Importantly, it has been reported that the reduction of macrophages in injured sites or depletion of macrophages impaired skeletal muscle regeneration after injury (30). Further, abrogated macrophage recruitment by C-C chemokine receptor type 2 deficiency resulted in the reduction of IGF-1 expression (46). IGF-1 is a hormone that has been considered as a biomarker of pathological conditions. Binding IGF-1 to IGF-1 receptor activates the PI3K/Akt-mTOR pathway, which stimulates protein synthesis and muscle maintenance. Lower IGF-1 levels in muscle are associated with inflammation (47). Macrophages are an important source of IGF-1, suggesting that macrophages have a pivotal ability in muscle protection and immune homeostasis (16, 48). Moreover, M1 macrophages inhibit myotube fusion by releasing TNF-α and IL-1β; however, M2 macrophages stimulate myotube formation by expressing high levels of IGF-1 and anti-inflammatory cytokines, such as IL-10 and TGF-β (17, 41). Thus, regulating the balance of M1 and M2 macrophages in muscle to prevent the progression of inflammation can be regarded as a novel therapeutic strategy for sarcopenia.

Current treatments for sarcopenia patients include nutritional supplements and hormone-related therapies that improve nutritional state, appetite, and total body mass. However, these treatments may increase the risk of cancer progression and have been reported to cause fluid retention, orthostatic hypotension, and hypogonadism (49–51). To cure or prevent sarcopenia without causing adverse effects, the drug should have low toxicity and be tested in cancer with a long-term schedule to determine whether it alters the efficacy of chemotherapies or promotes tumor growth. Although further investigation is needed to verify whether it is clinically relevant, here we demonstrated a protective effect of magnolol on cisplatin-induced muscle atrophy through the inhibition of inflammation in muscle without accelerating the tumor growth *in vivo*. In addition, we verified a novel role of magnolol in the phenotypic transition of macrophages. Thus, magnolol could be used with chemotherapeutic agents to prevent dose-limiting side effects in cancer patients.

## Data Availability Statement

All datasets generated for this study are included in the article/Supplementary Material.

## Ethics Statement

The animal study was reviewed and approved by the University of Kyung Hee Institutional Animal Care and Use of Committee.

## Author Contributions

CL performed the majority of experiments and wrote the manuscript. HJ, HL, MH, and SP contributed to data acquisition. HB designed the study.

### Conflict of Interest

The authors declare that the research was conducted in the absence of any commercial or financial relationships that could be construed as a potential conflict of interest.

## Supplementary Material

Supplementary Figure 1 Protective effect of magnolol in cisplatin-induced muscle atrophy was not dose dependent *in vivo*.**(A)** Body weight changes during the whole experiment, and **(B)** relative body weight vs. control (%) in various groups (magnolol: 1, 5, and 10 mg/kg). **(C)** The all-limbs grip strength measured by digital force gauge. **(D)** Representative images of hind limb muscles and **(E–G)** muscle mass of **(E)** TA, **(F)** EDL, and **(G)** SOL. **(H)** Histology of TA muscle stained with HandE (scale bar: 200 μm) and **(I)** diameter of cross-sectional muscle fibers. All data are expressed as the mean ± SEM of 5 mice. ^*^*P* < 0.05; ***P* < 0.01; ****P* < 0.001 vs. con and ^#^*P* < 0.05; ^*##*^*P* < 0.01; ^*###*^*P* < 0.001 vs. cis based on the one-way ANOVA Tukey's test.

Supplementary Figure 2 Gating strategy for analysis of macrophages in TA muscle tissues. TA muscles from wild type mice received vehicle (con) or 10 mg/kg magnolol (mag) were isolated and analyzed by flow cytometry. **(A)** Total cells were gated based on the FSC-A and SSC-A. **(B)** Only singlets were selected from the SSC-A vs. SSC-H dot plot. For Figure 4A, CD45 vs. IGF-1 cells were plotted gated on singlets. For Figure 4B, F4/80 expression was determined in CD45^+^IGF-1^+^ and CD45^−^IGF-1^+^ cells, and then the percentage of F4/80^+^ macrophages was compared. **(C)** After gating on CD45^+^ and CD11b^+^ cells, F4/80^+^IGF-1^+^ populations were compared for Figure 4C. **(D)** For Figures 4D,E, the percentage of CD11b^+^F4/80^+^ macrophages was determined after gating on CD45^+^ population and BrdU-labeled populations were analyzed within CD11b^+^F4/80^+^ macrophages. Red dots indicate isotype controls and black dots denote the stained cells with specific antibodies.

## Abbreviations

BMDM: bone marrow-derived macrophages
BrdU: bromodeoxyuridine
BUN: blood urea nitrogen
EDL: extensor digitalis longus
GAPDH: glyceraldehyde-3-phosphate dehydrogenase
IGF: insulin-like growth factor
IL: interleukin
iNOS: inducible nitric oxide synthase
LLC: Lewis lung carcinoma
LPS: lipopolysaccharide
M-CSF: macrophage colony-stimulating factor
MYHC: myosin heavy chain
NF-κB: nuclear factor kappa-B
PI3K: phosphoinositide 3-kinase
SOL: soleus
TA: tibialis anterior
TGF: transforming growth factor
TNF: tumor necrosis factor.
