# Supplementary figures and images for: Magnolol Attenuates Cisplatin-Induced Muscle Wasting by M2c Macrophage Activation
Source: Front Immunol. 2020 Feb 7;11:77. doi: 10.3389/fimmu.2020.00077 (PMC7018987; doi:10.3389/fimmu.2020.00077)

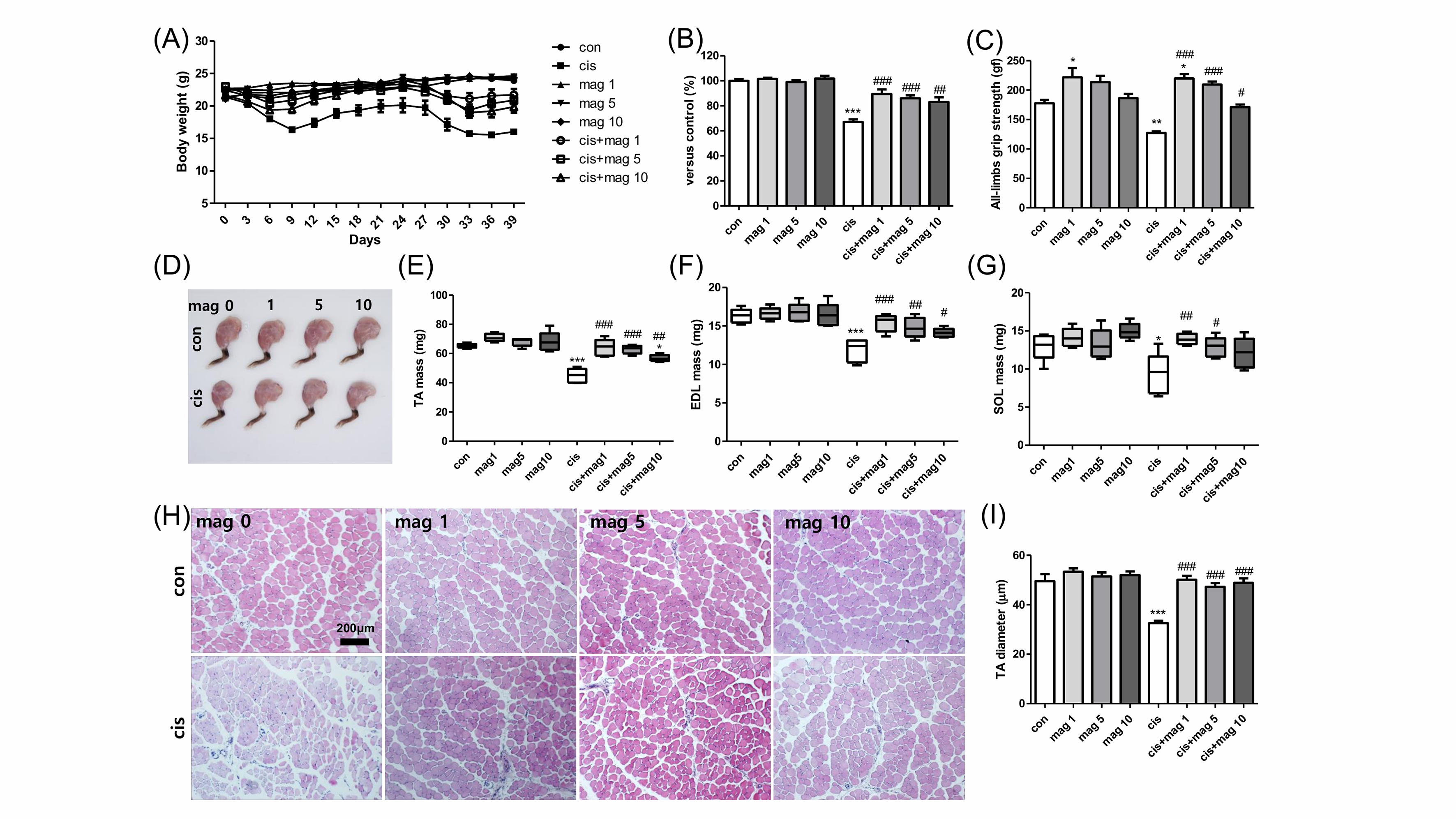

Supplement: Supplementary Figure 1 — Protective effect of magnolol in cisplatin-induced muscle atrophy was not dose dependent in vivo.(A) Body weight changes during the whole experiment, and (B) relative body weight vs. control (%) in various groups (magnolol: 1, 5, and 10 mg/kg). (C) The all-limbs grip strength measured by digital force gauge. (D) Representative images of hind limb muscles and (E–G) muscle mass of (E) TA, (F) EDL, and (G) SOL. (H) Histology of TA muscle stained with HandE (scale bar: 200 μm) and (I) diameter of cross-sectional muscle fibers. All data are expressed as the mean ± SEM of 5 mice. *P < 0.05; **P < 0.01; ***P < 0.001 vs. con and #P < 0.05; ##P < 0.01; ###P < 0.001 vs. cis based on the one-way ANOVA Tukey's test. [file Image_1.TIF]

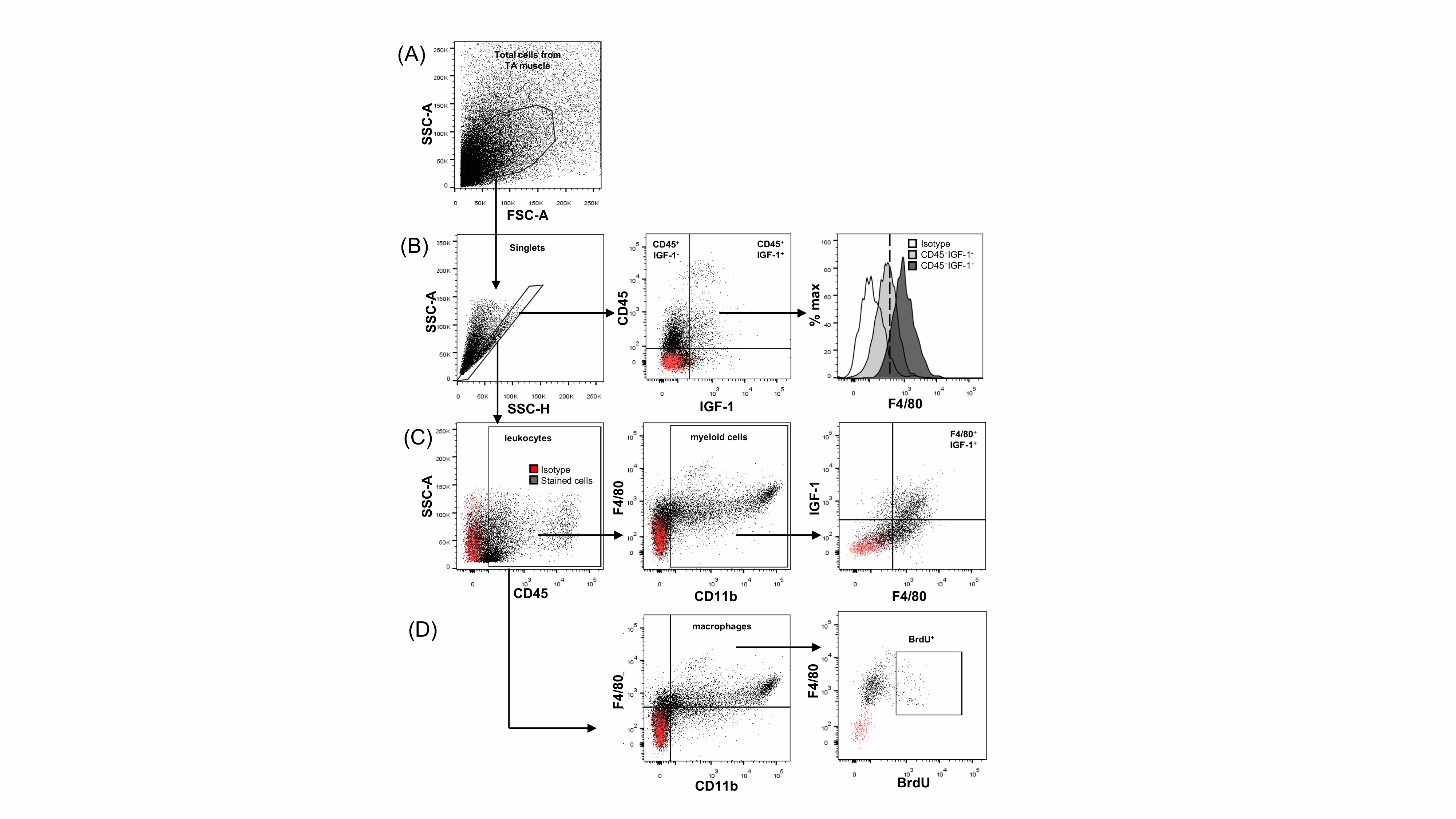

Supplement: Supplementary Figure 2 — Gating strategy for analysis of macrophages in TA muscle tissues. TA muscles from wild type mice received vehicle (con) or 10 mg/kg magnolol (mag) were isolated and analyzed by flow cytometry. (A) Total cells were gated based on the FSC-A and SSC-A. (B) Only singlets were selected from the SSC-A vs. SSC-H dot plot. For Figure 4A, CD45 vs. IGF-1 cells were plotted gated on singlets. For Figure 4B, F4/80 expression was determined in CD45+IGF-1+ and CD45−IGF-1+ cells, and then the percentage of F4/80+ macrophages was compared. (C) After gating on CD45+ and CD11b+ cells, F4/80+IGF-1+ populations were compared for Figure 4C. (D) For Figures 4D,E, the percentage of CD11b+F4/80+ macrophages was determined after gating on CD45+ population and BrdU-labeled populations were analyzed within CD11b+F4/80+ macrophages. Red dots indicate isotype controls and black dots denote the stained cells with specific antibodies. [file Image_2.TIF]
